# A Text-Book of Diseases of Women

**Published:** 1899-06

**Authors:** 


**A Text-Book of Diseases of Women.** By Charles B. Penrose, M.D. Second Edition. Pp. 529. London: The Rebman Publishing Co. (Ltd.). Philadelphia: W. B. Saunders. 1898.

This is an excellent work which goes straight to the mark, untrammelled by needless anatomical, pathological, and histological details. The recommendation of one plan of treatment for each disease is useful for didactic purposes, but is not without its drawbacks when we consider the various clinical modifications of disease. For example, the operation for vesico-vaginal fistula described on pp. 400—402, though sufficient for the majority of cases, is inapplicable in many others in which the sloughing has been very extensive. In this connection, however, we are glad to see that the author condemns kolpokleisis. Dr. Penrose's style is clear and incisive, and the book may be taken as a trustworthy exposition of modern gynæcology.

